# Effect of Tetrapropylammonium Chloride Quaternary Ammonium Salt on Characterization, Cytotoxicity, and Antibacterial Properties of PLA/PEG Electrospun Mat

**DOI:** 10.1002/bip.23626

**Published:** 2024-09-11

**Authors:** Sena Özdil Şener, Sema Samatya Yilmaz, Merve Dandan Doganci, Erdinc Doganci

**Affiliations:** ^1^ Science Institute, Department of Biomedical Engineering Kocaeli University Kocaeli Türkiye; ^2^ Engineering Faculty, Department of Chemical Engineering Kocaeli University Kocaeli Türkiye; ^3^ Department of Chemistry and Chemical Processing Technologies Kocaeli University Kocaeli Türkiye

**Keywords:** biodegradable, electrospinning, nanofiber, PLA nanofiber, quaternary ammonium salt

## Abstract

In this study, poly(lactic acid) (PLA)–tetrapropylammonium chloride (TCL)–poly(ethylene glycol) (PEG) nonwoven networks were produced using PLA, PEG with different concentrations (3, 5, 7, and 9 wt%), and TCL. PEG is included as a plasticizer in PLA polymer, which has high biocompatibility but a brittle structure. The importance of this study is to investigate the effect of TCL salt on the characterization of PLA–PEG nanofibers. For this research, the cytotoxicity test system responsible for the fibroblast cell line (L929) was evaluated with the liquid absorption capacity (LAC) and drying time tests for its use in wound dressings. The addition of TCL salt reduced bead formation in PLA–PEG nanofibers and increased the homogeneity of fiber dispersion. The smoothest and most homogeneous nonwoven networks were obtained as PLA–5TCL–PEG. It was also reported that this nonwoven network exhibited liquid absorption behavior with a maximum increase of 150% compared to the PLA–PEG nonwoven network and had the highest Young's modulus value of 12.97 MPa. In addition to these tests, evaluations were made with Fourier transform infrared spectroscopy (FTIR), scanning electron microscopy (SEM), drying time test, differential scanning calorimetry (DSC), thermogravimetric analysis (TGA), and mechanical tests. In addition, high cell viability was observed in L292 mouse fibroblast cells at the end of the 24th hour, again with the effect of TCL salt. In addition, antibacterial activity was tested against gram‐negative *E. coli* and gram‐positive *S. aureus* bacteria, and it was observed that there was no antibacterial activity. Since PLA–TCL–PEG nonwoven webs have a maximum cell viability of 133.27%, they are recommended as a potential dermal wound dressing.

## Graphical abstract

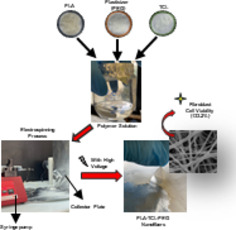

## 1 Introduction

The skin is the largest organ in the body and is responsible for protecting the skin tissue against external dangers. A wound is caused by an external tear that causes trauma to the skin and is frequently addressed in research because its healing is a complex process [1, 2, 3]. Wound dressings are used to protect the wound from the external environment, provide a moist environment around the wound, facilitate the healing of wounds, and shorten the healing time. Micro‐ and nanofibrous materials are suitable for the preparation of wound dressings. Today, many types of wound dressings have been developed to both prevent the wound from being affected by infection while it is healing and to support and accelerate the wound‐healing process. Recently, nanofibrous polymer materials prepared by the electrospinning method have attracted great attention due to their properties such as a high surface area/volume ratio, fine pores, and high porosity [4, 5]. In addition, it is generally used for exudative wounds due to its ability to absorb liquid, and thanks to its ability to retain exudate, it does not harm the wound during dressing. Studies have shown that nonwoven mesh electrospun scaffolds increase hemostasis, fluid absorption, cell respiration, and gas permeability when implanted into open wounds [6, 7]. These properties make nonwoven mats composed of electrospun fibers excellent candidates for a variety of applications such as wound dressings and drug delivery systems [8, 9, 10]. Furthermore, these nanofibrous networks can also promote cell adhesion, migration, growth, and differentiation, as well as angiogenesis, which are vital events for the emergence of an effective wound‐healing process [7].

It has been demonstrated in medical applications that the fiber diameter of nanofibers regulates the spread, orientation, and proliferation of osteoblastic cells, which are the main factors of temporary skin coverings or dressings [11, 12, 13]. As a bioactive polymer matrix, synthetic and natural polymers are widely prepared in nanofiber scaffolds for biomedical applications such as wounds [1, 14, 15, 16]. Polylactic acid (PLA), which is frequently preferred in biomedical applications due to its biodegradability and biocompatibility, is a polymer in the group of thermoplastic polyesters obtained from renewable resources (such as corn starch) and is not affected by moisture and water [17, 18].

Different biopolymers are used in studies conducted in the literature. These are natural polymers such as PLA, polycaprolactone (PCL), and polyvinyl alcohol (PVA), which have no toxic effects on living tissues and are biodegradable and self‐composting at the end of their useful life. Since they can be obtained from sustainable and renewable sources, they are widely used in biomedical fields such as scaffolds, artificial tissues, and wound dressings [19, 20, 21, 22, 23, 24, 25, 26, 27]. Ghosal et al. prepared biodegradable poly(ε‐caprolactone) nanofibers, collagen‐coated versions, and titanium dioxide–containing versions by electrospinning for skin tissue engineering and examined the surface and structural compatibility of these scaffolds [28]. Additionally, the plasticity and mechanical properties of polymeric films obtained by electrospinning were also discussed in a different article [29]. Lin et al. described the synthesis of novolac epoxy resin–modified polyurethane acrylates (EPUAs), creating a grafted polymer network that is different from traditional interpenetrating polymer networks [30]. Knunova et al. have discussed the development of antibacterial nanofibers using slow degradation of polycaprolactone (PCL) and halloysite nanotubes (HNTs) [31]. Despite its high tensile strength and modulus, there are studies in the literature where PLA is used with a suitable plasticizer to reduce the brittleness of its structure due to its low helix density and chain toughness [32, 33, 34]. Polyethylene glycol (PEG) is generally preferred as the plasticizer [35, 36, 37]. PEG is a plasticizing polymer that can be synthesized in a wide molecular weight range, is hydrophilic, flexible, can increase mechanical strength, and does not create toxic effects [17]. In addition, properties such as antibacterial activity and high cell viability can be increased in the products obtained, thanks to different additives (such as silver nanoparticles [38], chitosan [39], and curcumin [40]) added into polymer mixtures.

Quaternary ammonium salts (QAS) are considered the main class of cationic surfactants used in antistatic, fabric softeners, biocides, disinfectants, detergents, phase transfer agents, and many types of personal care product ingredients such as hair care products [41, 42, 43]. QAS is frequently the subject of studies due to its low cost and the benefits it brings to the structure [44, 45]. When QAS structures are examined, they contain at least one hydrophobic hydrocarbon chain attached to a positively charged nitrogen atom and other alkyl groups, which are often short‐chain substituents such as benzyl or methyl groups [46]. Tetrapropylammonium chloride TCL (C_12_H_28_ClN) is also known as a quaternary ammonium salt [47]. As a result of the literature research, there are very few studies on TCL salt, which makes this study, in which TCL salt will be evaluated with current characterization techniques, unique.

In this study, a wound dressing with the potential to provide rapid wound‐healing was designed with the addition of TCL. In this way, it is aimed to create a surface structure that will support cell adhesion and proliferation. Moreover, it was aimed to create a nanofiber structure that could absorb exudate fluid and support wound‐healing by keeping the wound environment moist. In order to evaluate the use of the obtained nanofibers as wound dressings, a mouse fibroblast cell line (L929) cytotoxicity test was performed. Liquid absorption capacity (LAC) and drying time tests of nanofibers designed as wound dressings were also performed. In addition, the antibacterial activity of TCL salt was investigated against gram‐negative and gram‐positive bacteria *Escherichia coli* and *Staphylococcus aureus*. The effect of TCL salt on the fiber properties of nanofibers obtained from PLA/PEG mixture was also examined. Detailed characterization of the obtained nanofibers was carried out by Fourier transform infrared spectroscopy (FTIR), and studies were carried out by Sscanning electron microscopy (SEM), differential scanning calorimetry (DSC), and thermogravimetric analysis (TGA).

## 2 Materials and Methods

### 2.1 Materials

PLA, containing roughly 4% by weight of the Luminity LX175 (Mw: 128.49) brand D‐isomer, was supplied by Total Corbion (Gorinchem, the Netherlands) and was used as the plasticizer for Fluka's PEG, a polymer with a molecular weight of 2000. Merck's tetrapropylammonium chloride (C_12_H_28_ClN) was used as the quaternary ammonium salt. ISOLAB's *N*,*N*‐dimethylformamide (DMF, Extra pure), Carlo‐Erba's dichloromethane (DCM, 99.9%), and J.T. Baker's chloroform (CF, 99%) are the solvents used in this research.

### 2.2 Preparation and the Electrospinning Process of PLA–PEG and PLA–TCL–PEG Solutions

Before preparing the polymer solution, the drying process was carried out under a vacuum at 55°C for 12 h to remove moisture from the PLA polymer. A literature search was conducted for the mixing ratio of PLA polymer in solution and it was determined as 7%wt [48, 49]. Due to the weak mechanics and brittleness of PLA, a PLA solution called PLA/PEG was prepared and used with the addition of 20% PEG polymer by weight for nanofiber production [50]. TCL salt was added to the matrix at ratios of 3, 5, 7, and 9%wt, depending on the amount of solid PLA in the solution. The prepared solution was dissolved with a DCM/DMF/CF (7v/2.5v/0.5v) solvent mixture in an airtight beaker for 3 h with the help of a magnetic stirrer. All prepared solutions were subjected to the electrospinning process without waiting in the same way. Electrospinning parameters were kept constant at 17 kV, 15 cm distance between syringe and plate, and 1.5 mL/h feeding rate. Environmental conditions during production were measured as approximately 40% relative humidity and 25°C. The resulting nonwoven webs were identified according to the codes specified in Table 1.

**TABLE 1:** Codes of prepared solutions.

Codes of studies	PLA/PEG (wt, %)	TCL (wt/%)
PLA–PEG	80/20	—
PLA–3TCL–PEG		3
PLA–5TCL–PEG		5
PLA–7TCL–PEG		7
PLA–9TCL–PEG		9

### 2.3 Characterization of PLA–PEG and PLA–TCL–PEG Nonwoven Webs

IR spectra of PLA–PEG and PLA–TCL–PEG nonwoven webs were examined with a Perkin Elmer Spectrum 100 FTIR device with an ATR unit spectrometric feature in the range of 650–4000 cm^−1^.

The surface images of PLA–PEG and PLA–TCL–PEG nonwoven webs obtained in the study were characterized using a JEOL brand JSM‐6060 model scanning electron microscopy. The average fiber diameter of the nanofibers was measured using Image J software on SEM images.

The LAC % of PLA–TCL–PEG nanofibers was evaluated based on EDANA 10.3.99 guideline steps [51] with specific environmental conditions. The drying time test was carried out according to the literature [52]. LACs of nonwoven webs were evaluated using the following equation (Equation 1):

(1) $$\mathrm{LAC}\;\left(\%\right)=\frac{N_{s}-N_{k}}{N_{k}}\times 100$$

In the equation here, *N*_*k*_ is the initial dry weight of the nanofibers, and *N*_*s*_ represents the weight of the wet nanofibers. LAC tests were repeated 5 times for each web sample, and the values obtained at the end of each round were averaged and reported.

DSC analysis of nonwoven webs was performed in a single step with a Mettler Toledo DSC 1 device in the temperature range of 25 to 300°C with a heating rate of 10°C/min. During DSC analysis, high‐purity nitrogen gas was fed at a flow rate of 30 mL/min. The degree of crystallinity (*X*_*c*_) of composite electrospun mats was calculated according to “Equation (2)” below with the data obtained from DSC results.

(2) $$X_{c}\left(\%\text{crystallinity}\right)=\frac{∆H_{m}-∆H_{c}}{\left(\omega_{f}\right)\times ∆{H_{m}}^{0}}$$

In the equation here, *ΔHm* ^*0*^ represents the melting heat of the matrix. The *ΔHm* ^*0*^ value of the PLA (matrix) used in this study is 93.7 J/g [53]. *ΔH*_*m*_ is the heat and *ωf* is the weight rate of melting of each mat. TGA of all nonwoven webs was performed on a Mettler Toledo TGA 1 instrument with a temperature range of 25 to 600°C. During TGA analysis, the test was fed with high‐purity nitrogen gas at a flow rate of 30 mL/min, as in DSC.

The mechanical properties of nonwoven webs were characterized with a Lloyd Instruments LRX Plus brand tensile device by applying the ASTM D882 standard. During the test, the application was carried out at a load of 5 kN and a tensile speed of 1:10 mm/min. Each web surface was prepared for testing by cutting five samples in 50 × 15 mm dimensions following the standard. The average tensile test results of PLA–PEG and PLA–TCL–PEG nonwoven webs were calculated and recorded for evaluation.

For cytotoxicity testing using the direct contact method with WST‐8, mouse fibroblast cells (L929, ATCC, CCL‐1) were employed [54]. The L929 cell line was obtained through funding of our project from the American Type Culture Collection (ATCC) (Product code: CCL‐1). Since L929 cells are frequently used as cell lines in cytotoxicity studies and were obtained from ATCC, no test or authentication was performed. The cell viability (%) results obtained from cytotoxicity were calculated using the following equation (Equation 3):

(3) $$\text{Cell vailabilty}\%=\frac{A_{b}\;\text{sample}-A_{b}\;\text{blank}}{A_{b}\;\text{control}-A_{b}\;\text{blank}\;}\times 100$$

The absorbance of the sample is denoted as *A*_*b*_ *sample*, the absorbance of the blank is denoted as *A*_*b*_ *blank*, and the absorbance of the negative control is denoted as *A*_*b*_ *control*.

Agar plate colony counting was performed for 24 h using *E. coli* ATCC 8739 and *S. aureus* ATCC 6538P following the AATCC‐100 standard [52]. This procedure serves as a quantitative method to evaluate the antibacterial activity of PLA–TCL–PEG nonwoven webs. The antibacterial activity of nanofibers was evaluated using the following equation (Equation 4):

(4) $$\text{Antibacterial activity}\;\left(\%\right)=\left(A-B\right)/A\times 100$$

*A* represents the average colony count of the untreated mat designated as the control sample and *B* represents the average number of colonies per hour, excluding the count at hour 0, which has the fewest colonies.

## 3 Result and Discussion

### 3.1 Chemical Properties of PLA–PEG and PLA–TCL–PEG Nonwoven Webs by FTIR Analysis

**FIGURE 1:**
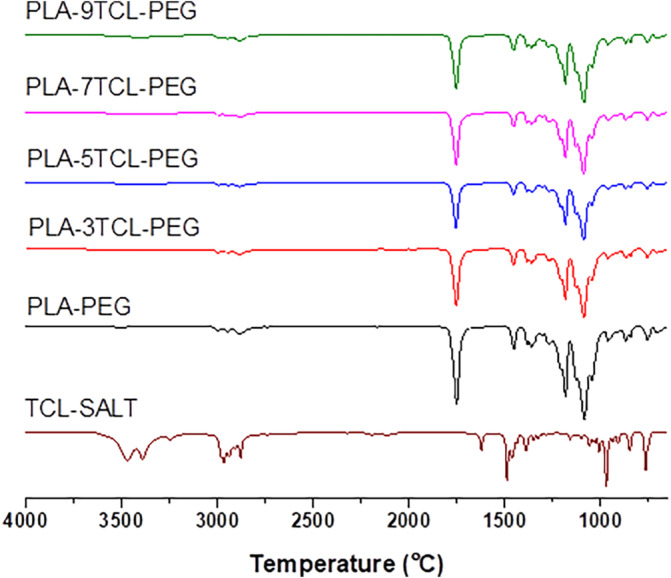
FTIR spectra of TCL salt, raw PLA–PEG, and PLA–TCL–PEG nonwoven webs.

FTIR spectra of TCL salt, PLA–PEG, and PLA–TCL–PEG nonwoven webs are given in Figure 1. When the FTIR spectrum of PLA/PEG nanofibers was examined, the prominent peak at 1753 cm^−1^ attributed to PLA revealed the presence of the (C=O) group, [52] while the peak at 1183 cm^−1^ (C—O—C) indicated the presence of strong ester groups [55]. The peak observed at 1271 cm^−1^ was identified as originating from the (C—O) group, which showed the presence of PLA within the material. Furthermore, the peak at 1453 cm^−1^ corresponded to the CH_3_ bending vibration associated with the PEG's molecular structure [56]. The distinctive peak at 1086 cm^−1^, characteristic of PEG, confirmed the presence of the (C—O) group [57]. Moreover, the peaks at 962 and 844 cm^−1^ were associated with the (–CH=CH–) group [58, 59], while the peak at 868 cm^−1^ was attributed to the (C—C) group bonding [60]. Notably, all these peaks characteristic of the PLA–PEG matrix were evident in the FTIR spectra of the PLA–TCL–PEG nanofibers.

The absence of an OH group in the structure of TCL showed that the broad stretching peak at 3471 cm^−1^ may be caused by moisture in the environment. All components from 2980 to 2800 cm^−1^ expressed asymmetric and symmetric stretching of sp^3^–CH_2_ and–CH_3_. In addition to the stretching vibration, the bending of sp^3^ –CH_2_ and –CH_3_ can also be observed in the region between 1465 and 1375 cm^−1^ [61].

Although it was not present in the spectrum of the TCL salt, it was observed that the peak at 1753 cm^−1^ in the PLA–PEG nonwoven webs visibly decreased in the PLA–TCL–PEG nonwoven webs with the addition of TCL salt. It has been stated that the web with the smallest peak intensity at 1753 cm^−1^ is PLA–5TCL–PEG. The 1622 cm^−1^ peak, which is the N—H deformation of the primary amine, [62] observed in the spectrum of the pure TCL salt, was clearly seen in the PLA–7TCL–PEG and PLA–9TCL–PEG networks. The intensity of the 1453 cm^−1^ peak seen in the pure PLA spectrum decreased slightly with the addition of TCL salt to the matrix. It was stated that the PLA–5TCL–PEG web had the smallest peak intensity at 1453 cm^−1^. The peak seen at 1183 cm^−1^ in the PLA–PEG nanofiber was observed to decrease with the addition of salt in the PLA–TCL–PEG webs. It was stated that the peak intensity of the peak seen at 867 cm^−1^ in the PLA–TCL–PEG web decreased compared to the pure PLA–PEG web and remained constant in all PLA–TCL–PEG webs. The peak at 840 cm^−1^ appeared similarly. It was stated that only the 840 cm^−1^ peak in the PLA–9TCL–PEG nonwoven web appeared more prominent than the others. It was observed that the peak at 840 cm^−1^ had the lowest peak intensity in the PLA–7TCL–PEG nonwoven web.

In line with these results, although no specific effect was observed with the addition of TCL salt to the PLA–PEG matrix, it was reported that it slightly affected the peak intensities in PLA–TCL–PEG webs. It has been documented that peak intensities vary depending on the location of salt ions within the polymer matrix and their chemical interactions with it.

### 3.2 Surface View Properties of PLA–PEG and PLA–TCL–PEG Nonwoven Webs by SEM

**FIGURE 2:**
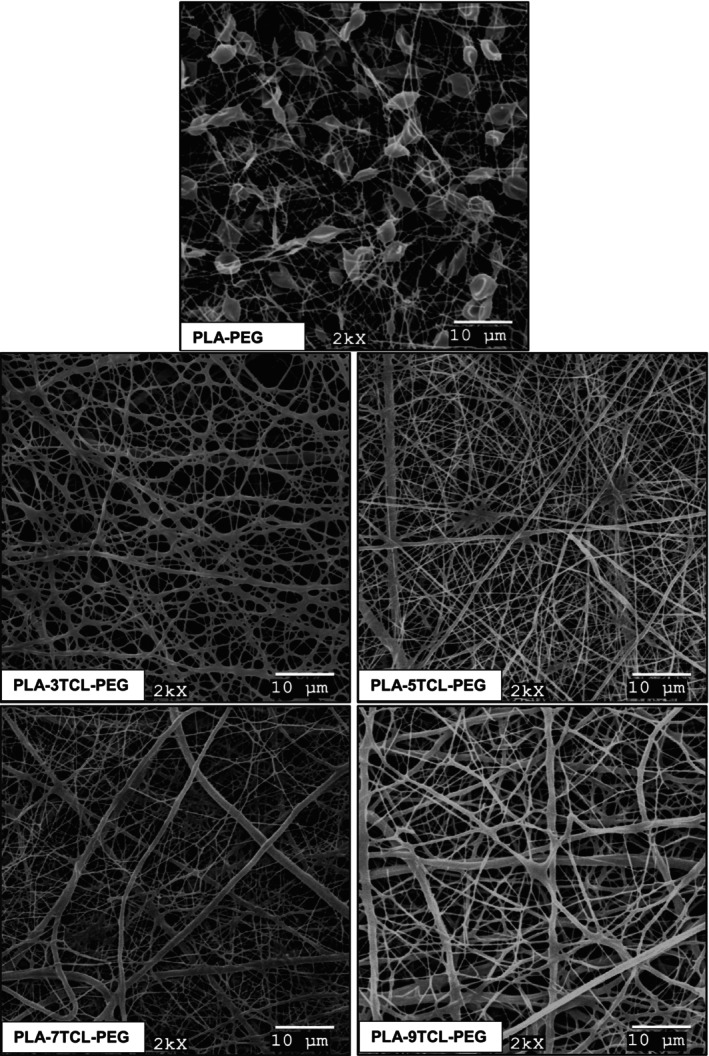
SEM images of raw PLA–PEG and PLA–TCL–PEG nonwoven webs (magnification: 2000×, scale: 10 μm).

**FIGURE 3:**
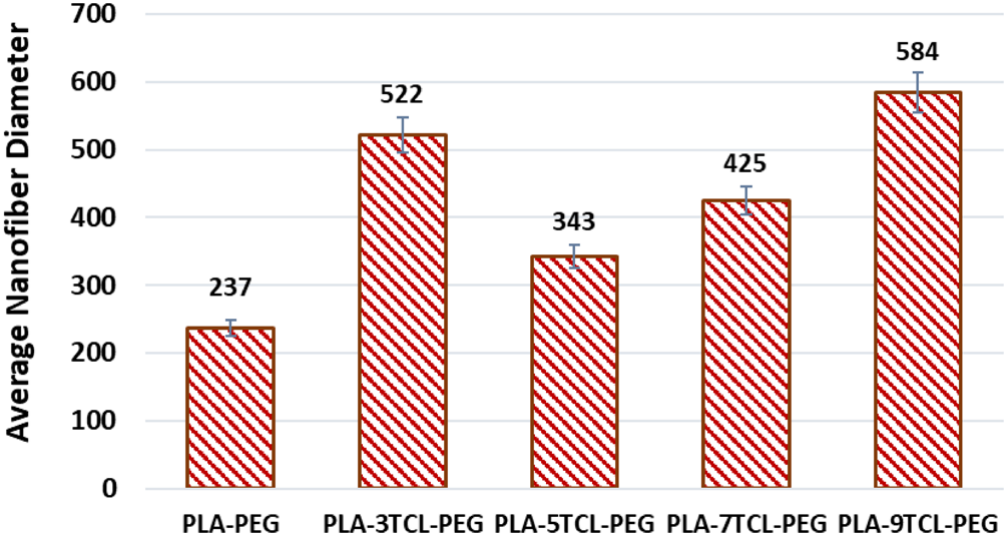
Average nanofiber diameter of raw PLA–PEG and PLA–TCL–PEG nonwoven webs.

Surface SEM images and average fiber diameter values of PLA–TCL–PEG nonwoven webs are given in Figures 2 and 3, respectively. It has been observed that pure PLA–PEG nanofiber has a bead structure. Although pure PLA–PEG nonwoven web has a beaded structure, it is stated that it has the lowest average fiber diameter with a value of 237 nm. It has been reported that, as a result of the addition of TCL salt to the PLA–PEG matrix, the beads seen in the structure decrease, and thus smooth nanofibers are obtained. While the solution viscosity increased with the addition of TCL salt to the PLA–PEG polymer matrix, the conductivity of the solutions also increased due to the presence of freely circulating salt ions in the environment [63]. The increase in solution viscosity also increased the surface tension. However, it has been observed that the fibrils in the 3% TCL‐added nonwoven web tend to stick together. The reason for this is that while the solution conductivity increases, the viscosity and surface tension of the solution also increase so that not enough voltage is used to produce smooth nanofiber surfaces. Because electrospinning application conditions are kept constant in all nonwoven web productions in order not to cause variability in nanofiber properties. It has been stated that PLA–5TCL–PEG nonwoven web has the most homogeneous, linear, and smoothest nanofibers. This result was associated with the fact that constant voltage, increasing solution viscosity, and conductivity constitute the most optimized parameters to produce PLA–TCL–PEG nanofibers. Besides, it was stated that among TCL salt‐doped nanofibers, PLA–5TCL–PEG nonwoven web had the lowest average fiber diameter with a value of 343 nm. Some thickening was observed in the nanofibers on the PLA–7TCL–PEG surface, and the average fiber diameter was measured as 425 nm. It has been reported that the nanofibers on the PLA–9TCL–PEG surface are quite thick and prone to adhesion, similar to the PLA–3TCL–PEG nonwoven web. The average fiber diameters of PLA–3TCL–PEG and PLA–9TCL–PEG nonwoven webs were measured as 522 and 584 nm, respectively. This reason was explained by the fact that the constant voltage is not strong enough to overcome the surface tension that increases with the increase in solution viscosity and conductivity. It has been reported that the optimum TCL salt addition to the PLA–PEG solution is 5%. It has been observed that with 7% TCL salt addition, the nanofiber surfaces thicken and are prone to adhesion.

### 3.3 Surface Properties of PLA–PEG and PLA–TCL–PEG Nonwoven Webs by LAC (%) and Drying Time (min) Analysis

**FIGURE 4:**
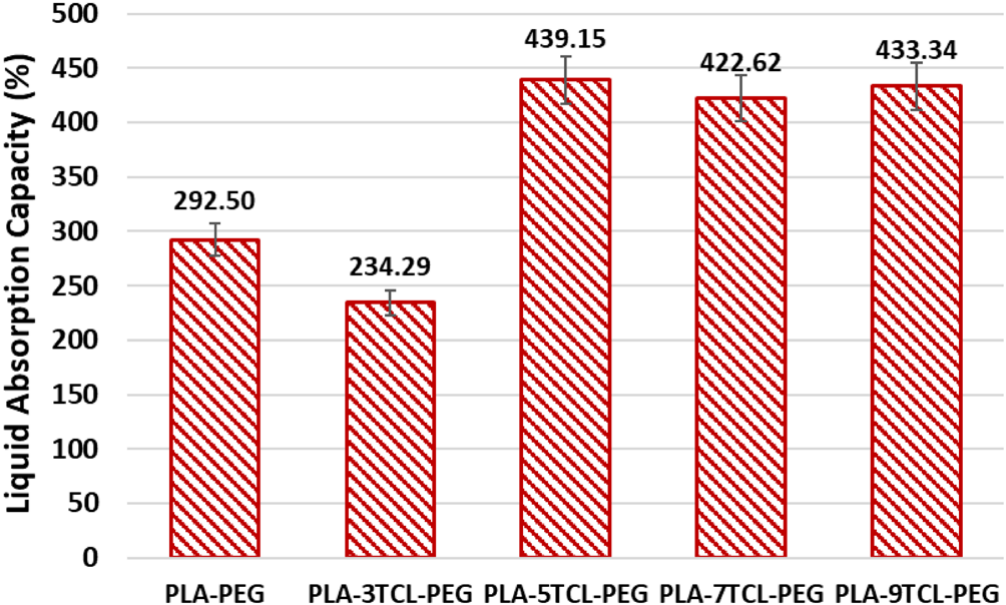
The liquid absorption capacity (%) of raw PLA–PEG and PLA–TCL–PEG nonwoven webs.

LAC % values of PLA–PEG and PLA–TCL–PEG nonwoven webs are given in Figure 4, and drying times (min) are given in Table 2. According to the liquid absorption test results, the PLA–PEG nonwoven web was reported as 292.5%. While PLA tends to repel water due to its hydrophobic structure, PEG tends to attract water or moisture due to its hydrophilic structure [17, 18]. Therefore, as a result of this tendency, the LAC of the PLA–PEG electrospun web is associated with PEG. With the addition of TCL salt to the PLA–PEG matrix, there was a visible increase in the absorption capacity of nonwoven webs except PLA–3TCL–PEG. The nature of salt's ability to hold water molecules is explained by its hygroscopic structure [64].

**TABLE 2:** The raw PLA–PEG and TCL‐based PLA–PEG nonwoven webs' average drying time with a standard deviation.

Samples	Average drying time	Standard deviation
PLA–PEG	5.00	1.00
PLA–3TCL–PEG	7.00	1.41
PLA–5TCL–PEG	3.50	0.70
PLA–7TCL–PEG	4.33	0.58
PLA–9TCL–PEG	5.50	0.70

It is thought that the first condition that affects the LAC ability of PLA–3TCL–PEG nonwoven webs is the fiber diameter thickness. It has been stated that in the presence of thick fibers, water will not be able to find a way to penetrate the structure, causing the LAC value to decrease. In this case, it can be said that 3% TCL salt addition does not affect LAC ability. When the contribution rate increased to 5%, the highest LAC value was obtained as 440.48%. It can be said that TCL salt was attracted to the water molecules in the environment because the salt distribution on the smooth fibers was the most homogeneous. It was observed that the LAC value of PLA–7TCL–PEG and PLA–9TCL–PEG nonwoven webs was significantly higher than that of pure PLA–PEG and 3% TCL salt‐added PLA–PEG samples. It has been reported that LAC values increase due to their thicker diameter fibers than PLA–5TCL–PEG nanofiber and the high amount of hydrophilic TCL in their structure.

According to the drying test results, PLA–PEG nonwoven web exhibited drying behavior in 5 min. However, with the addition of salt to the structure, the drying time first increased and then decreased. PLA–3TCL–PEG nonwoven web showed the highest drying time of 7 min. Even though it showed the lowest LAC value, very thick fibers are difficult to dry in a short time. It was noticed that the PLA–5TCL–PEG nanofiber, which had the highest LAC value, dried after 3.5 min. The fibers are thin and there are many air spaces. In this way, the necessary qualities for the fibers to dry in a short time are provided. PLA–7TCL–PEG and PLA–9TCL–PEG webs showed longer drying time compared to PLA–5TCL–PEG nanofiber, with values of 4.33 and 5.5 min, respectively. The longer drying time of these nanofibers than the PLA–5TCL–PEG nonwoven web is associated with the high amount of TCL salt ions in the structure taking up water. The LAC value of the PLA–9TCL–PEG web remained above the LAC value of the PLA–PEG nonwoven web, which is similar to that of the PLA–3TCL–PEG nanofiber. The PLA–9TCL–PEG web has a high amount of TCL salt in its structure and a high fiber diameter and thickness. The stated reasons caused the material to dry longer.

### 3.4 Thermal Properties of PLA–PEG and PLA–TCL–PEG Nonwoven Webs

Thermal properties of PLA–TCL–PEG nonwoven webs were determined by DSC and TGA, and the temperature values of the thermal properties are shown in Table 3.

**TABLE 3:** Thermal properties of PLA–PEG and PLA–TCL–PEG nonwoven webs obtained from DSC and TGA curves.

Samples	*T*_m1_ (°C)^a^	*T*_m2_ (°C)^b^	ΔH (J/g)^c^	*T*_m3_ (°C)^d^	*X*_c_ (%)^e^	*T*_5_ (°C)^f^	*T*_10_ (°C)^f^	*T*_max1_ (°C)^g^	*T*_max2_ (°C)^g^	Residue (%)^h^
PLA–PEG	52.6	156.4	28.5	—	29.8	300.1	308.9	—	338.2	7.06
PLA–3TCL–PEG	47.7	152.9	22.8	194.7	30.4	295.5	311.9	197.4	341.6	7.13
PLA–5TCL–PEG	48.9	154.4	22.1	195.2	29.5	261.1	288.4	196.9	330.3	7.39
PLA–7TCL–PEG	47.9	159.0	22.2	197.2	29.6	209.9	290.0	199.0	339.1	7.34
PLA–9TCL–PEG	53.4	159.0	21.6	196.4	28.8	204.1	287.5	199.0	341.2	7.40

^a^ *T*_m1_ gives the melting point of polymers according to PEG during the first heating process in DSC experiments.

^b^ *T*_m2_ gives the melting point of polymers compared to PLA in the second heating process in DSC experiments.

^c^ Enthalpy value of *T*_m2_ temperature in DSC experiments.

^d^ *T*_m3_ gives the melting point of polymers compared to TCL in the third heating process in DSC experiments.

^e^ The crystallinity is calculated according to the results obtained in DSC experiments.

^f^ *T*_5_ and *T*_10_ are the temperatures of 5% and 10% weight loss in the TGA test, respectively.

^g^ *T*_max1_ and *T*_max2_ temperatures *T*_max1_ and are the maximum degradation temperatures obtained from the derivative of the TGA curve.

^h^ The percent of the amount of residue at 600°C in TGA experiments.

**FIGURE 5:**
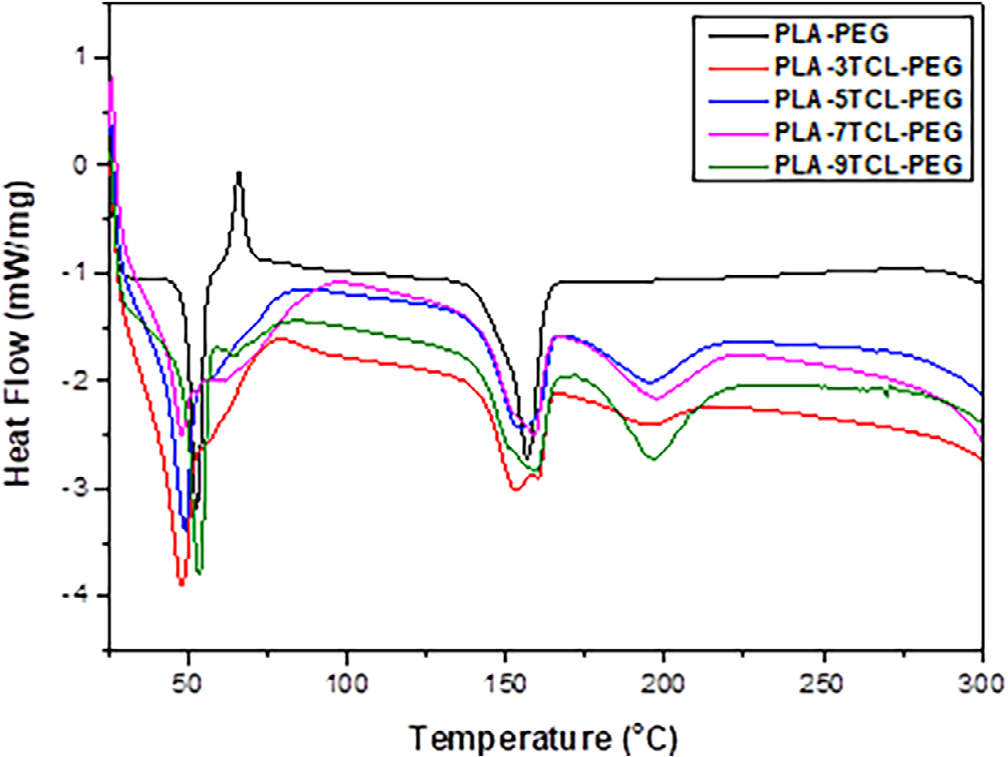
DSC curves of raw PLA–PEG and PLA–TCL–PEG nonwoven webs.

DSC curves of TCL salt and all PLA–PEG nonwoven webs are given in Figure 5. In the literature, the *T*_*g*_ temperature of pure PLA nanofibers has been reported as approximately 63°C, the *T*_*c*_ temperature as approximately 115°C, and the *T*_*m*_ temperature as approximately 152°C [33, 65]. Similarly, in the literature, the *T*_*c*_ temperature of pure PEG nanofiber is stated as 27.2°C and the *T*_*m*_ temperature is 55.6°C [66]. According to the obtained DSC test results, the melting temperatures of the TCL salt were measured as 67, 116, and 235°C.

In the measurements of pure PLA–PEG nanofibers, it was found that the *T*_*m*1_ and *T*_*m*3_ temperatures were 52 and 156°C, respectively, and the T_c_ crystallization temperature was 66°C (the ΔH value was 6.14 J/g) and its crystallinity was 29.8%. By adding TCL salt, *T*_*m*1_ temperature was obtained in the range of 47–53°C. It has been stated that the PLA–PEG nonwoven web is the closest to the *T*_*m*1_ temperature of the PLA–9TCL–PEG nonwoven web. The effect of the melting temperature of pure TCL salt on the matrix was noticed with the emergence of *T*_*m*3_ temperature in PLA–TCL–PEG nonwoven webs. The *T*_*m*3_ temperature seen in PLA–TCL–PEG nonwoven webs was measured in the range of 194–197°C. Since no visible change was noticed in the crystallinity values, it was evaluated that the TCL salt additive had no effect on crystallinity.

**FIGURE 6:**
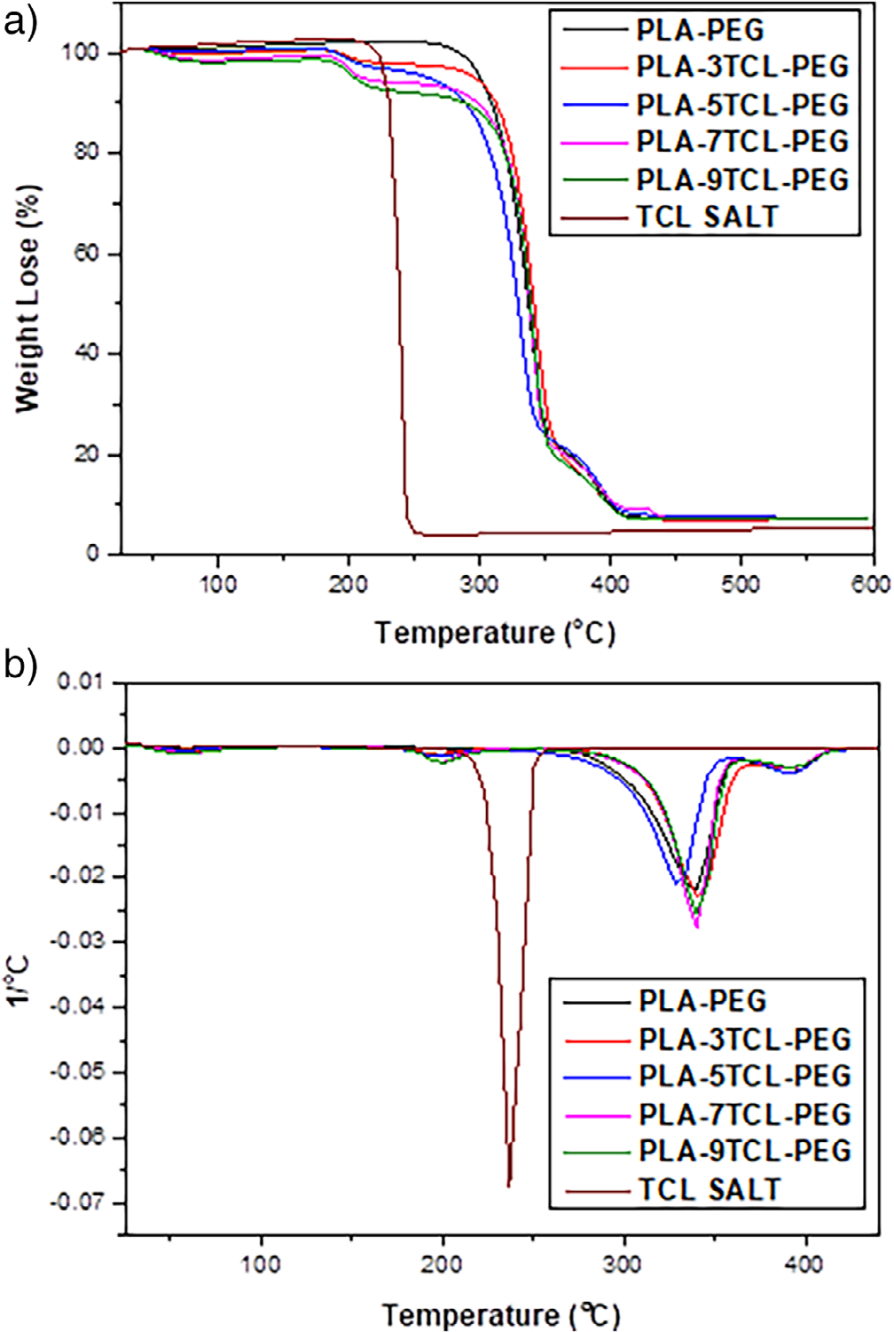
(a) Thermal decomposition curves and (b) derivatives of the thermal decomposition curves of TCL salt.

The TGA curves and derivates of PLA–PEG and PLA–PEG‐TCL nonwoven webs are shown in Figure 6a,b. TCL salt showed one‐step decomposition behavior and its decomposition temperature was reported to be 237.2°C. The PLA–PEG nonwoven web exhibited a two‐step degradation behavior at temperatures of 338.3 and 390.7°C. When these obtained values were compared with the literature, they were associated with the characteristic degradation temperature values of pure PLA and PEG [67, 68]. *T*_max1_ values seen in PLA–TCL–PEG nonwoven webs arise from the characteristic decomposition temperature of pure TCL salt. The *T*_max2_ temperature was correlated with the degradation temperatures of PLA–PEG nonwoven webs. A decrease in the *T*_deg5_ temperature of PLA–3TCL–PEG nonwoven webs was reported with the increase in the TCL salt ratio added to the structure. While no change was observed in the PLA–3TCL–PEG nanofiber for *T*_deg10_ temperature, a decrease was detected with the increase in the amount of TCL salt. When the amount of residue of nonwoven webs was examined, a slight increase was reported with the addition of TCL salt.

### 3.5 Mechanical Properties of PLA–PEG and PLA–TCL–PEG Nonwoven Webs by the Tensile Test

**FIGURE 7:**
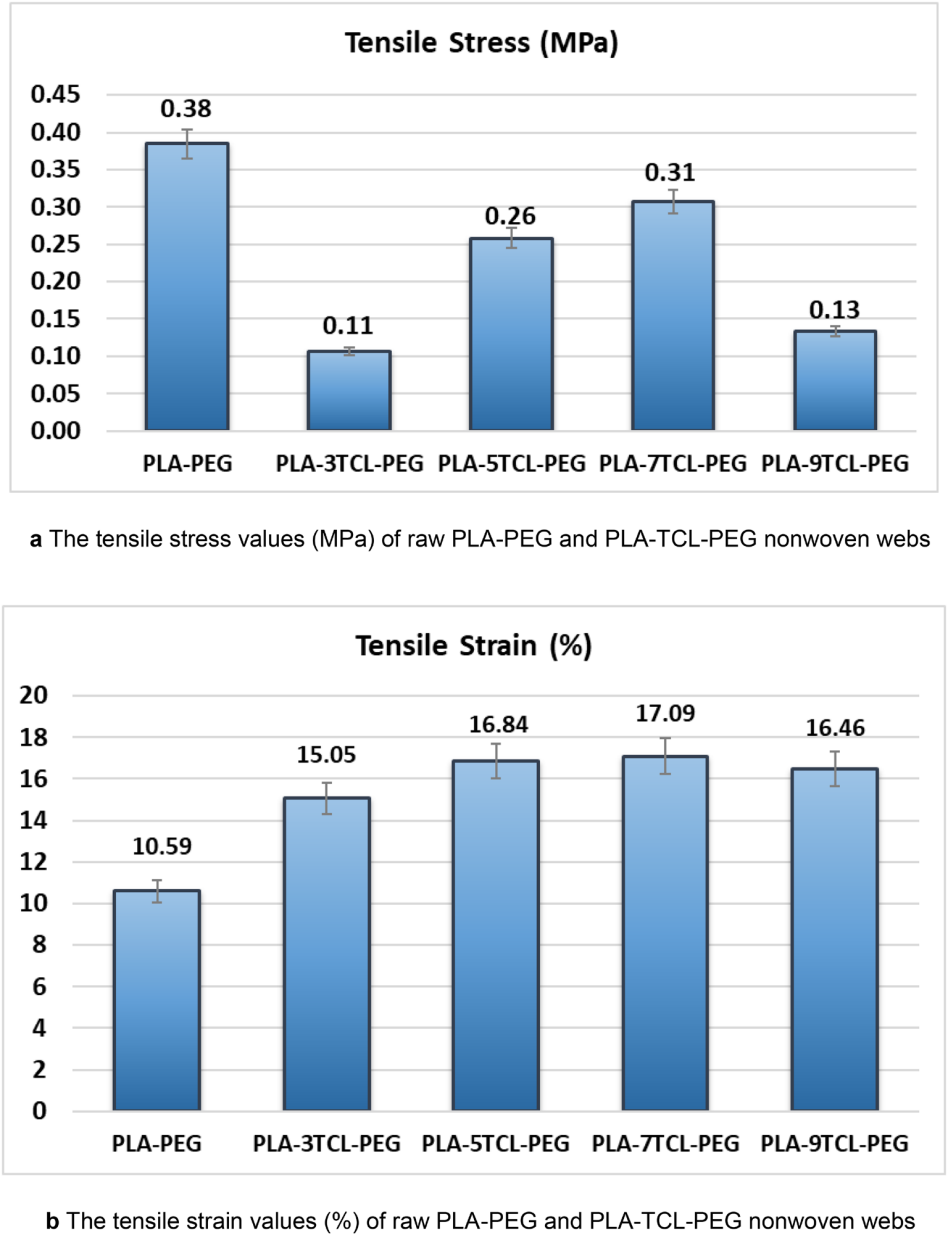
(a) The tensile stress values (MPa) of raw PLA–PEG and PLA–TCL–PEG nonwoven webs. (b) The tensile strain values (%) of raw PLA–PEG and PLA–TCL–PEG nonwoven webs.

**FIGURE 8:**
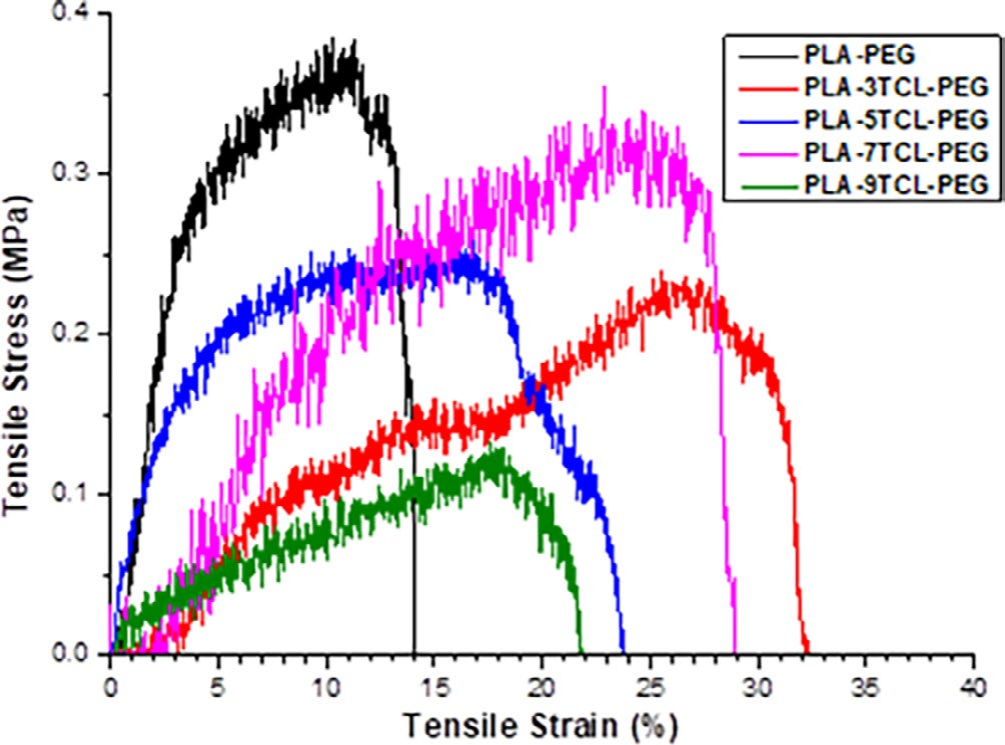
The stress–strain curves of raw PLA–PEG and PLA–TCL–PEG nonwoven webs.

Tensile stress and tensile strain graphs of pure PLA–PEG and PLA–TCL–PEG nonwoven webs are given in Figure 7a,b, respectively. At the same time, tensile stress–strain curves are shown in Figure 8. It was observed that pure PLA–PEG nonwoven web gave the highest tensile strength with a value of 0.38 MPa. A decrease in the tensile strength of the nanofibers was seen after the addition of TCL salt. A sharp decrease in the tensile strength of PLA–3TCL–PEG and PLA–9TCL–PEG nonwoven webs, especially with 3% and 9% additives, was noticeable. This situation was attributed to the irregular distribution of salt molecules on the polymer chain. The tensile strength of 5% and 7% TCL salt‐added nanofibers was found to be slightly closer to that of pure PLA–PEG nanofibers. PLA–5TCL–PEG and PLA–7TCL–PEG nonwoven webs exhibited tensile strengths of 0.26 and 0.31 MPa, respectively. When the tensile strain behavior of nonwoven webs was examined, it was stated that pure PLA–PEG nanofiber showed the lowest elongation with a value of 10.59%. With the addition of TCL salt, the tensile strain values of all nanofibers increased. It has been observed that PLA–TCL–PEG nanofibers have almost the same tensile strain values. In particular, it was stated that the PLA–7TCL–PEG nonwoven web exhibited the highest tensile strain value with 17.09%.

Among TCL salt‐doped nanofibers, nonwoven webs showing high tensile strength and high tensile strain values have been reported to be PLA–5TCL–PEG and PLA–7TCL–PEG, respectively. It was stated that 5% and 7% TCL salt additives added to the pure PLA–PEG matrix were sufficient to improve the mechanical properties of the material.

**FIGURE 9:**
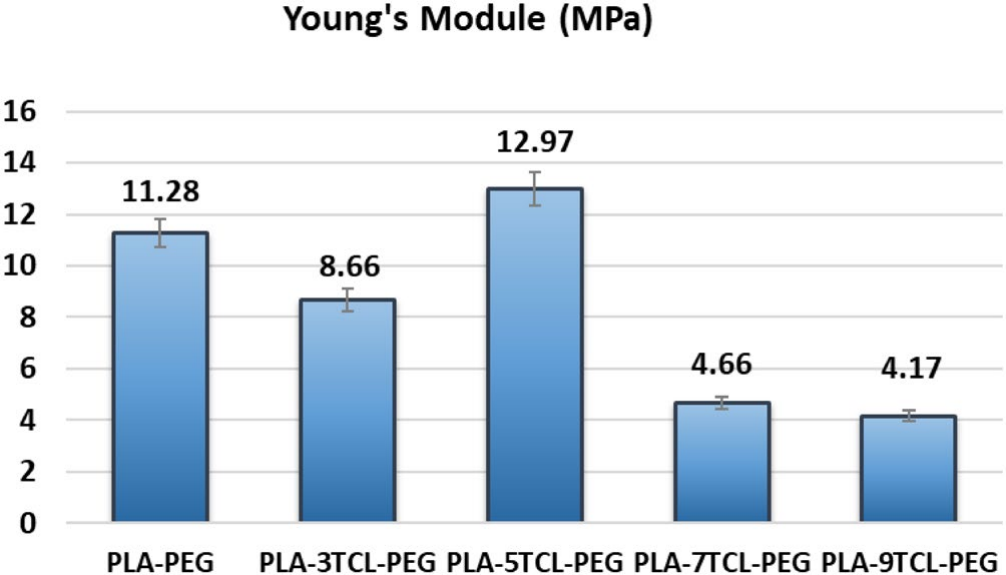
Young's Modulus values (MPa) of raw PLA–PEG and PLA–TCL–PEG nonwoven webs.

When the tensile stress–tensile strain curves of nonwoven webs were examined, it was stated that the toughness behavior of 7% TCL salt‐added PLA–7TCL–PEG nanofiber was more advanced than that of other nanofibers. It was emphasized that although it has the highest tensile strength, the most brittle material is the pure PLA–PEG nonwoven web and exhibits the lowest ductile behavior. According to the SEM images of the pure PLA–PEG nanofiber, it has been stated that the beads on the surface increase the tensile strength while decreasing the elongation [52]. It was also stated that the nonwoven web with the lowest toughness was PLA–9TCL–PEG. Young's modulus values of pure PLA–PEG, and PLA–TCL–PEG nonwoven webs are shown in Figure 9. Young's modulus values confirm the toughness and ductility behavior of nanofibers. While the PLA–9TCL–PEG nanofiber showed the lowest Young's modulus value, the PLA–5TCL–PEG nanofiber exhibited the highest Young's modulus. It has been associated with the fact that crystal salt molecules distributed irregularly on the polymer chain reduce the modulus of the structures.

### 3.6 Cytotoxicity Testing and Antibacterial Activity of PLA–PEG and PLA–TCL–PEG Nonwoven Webs

**FIGURE 10:**
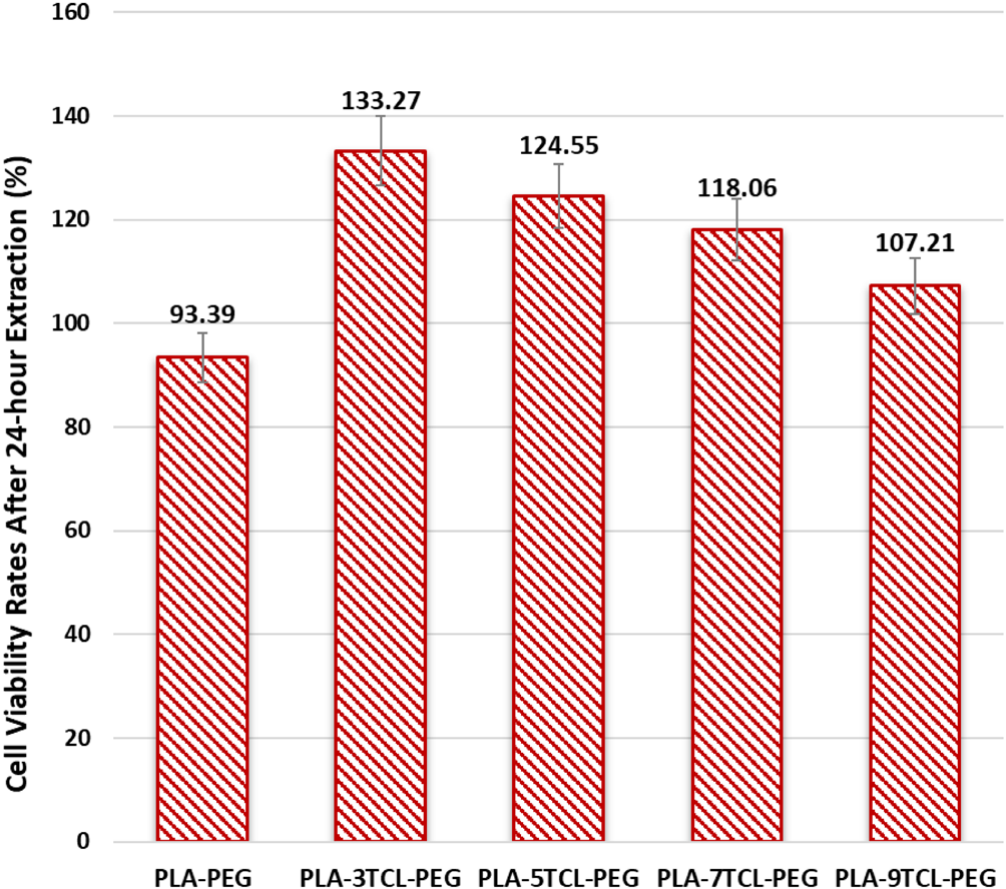
Cell viability (%) of raw PLA–PEG and PLA–TCL–PEG nonwoven webs.

The cytotoxicity test results determining the cell viability (%) of PLA–TCL–PEG nonwoven networks are shown in Figure 10. Among all nonwoven networks, the PLA–PEG nonwoven network exhibited the lowest cell viability value at 93.39% at the end of the 24th hour. The presence of a beaded structure in the PLA–PEG nanofiber surface image confirmed this result. The beaded structures seen on the surface prevented cell growth [69]. A significant increase in cell viability values of PLA–TCL–PEG nanofibers was observed with the addition of TCL salt to the matrix. Among the salt‐doped electrospun networks, the highest cell viability was seen in the PLA–3TCL–PEG nanofiber with 133.27%, and a gradual decrease was observed as the rate of addition of the salt additive to the PLA–PEG matrix increased. The smoothest and lowest‐diameter fibers appeared on the surface of the PLA–5TCL–PEG nonwoven network, which facilitated the adhesion and proliferation of cells. Although cell viability decreased slightly compared to the 3% TCL salt contribution, the cell viability value of the PLA–5TCL–PEG electrospun network was found to be 124.55%. The presence of thick fibers in the PLA–7TCL–PEG nonwoven web reduced the cell viability to 118.06%. Although the cell viability value of the PLA–9TCL–PEG network represents the lowest cell viability among PLA–PEG nanofibers with 107.21%, it is still stated to have a high value compared to pure PLA–PEG. This result is related to the fact that PLA–9TCL–PEG nonwoven fabric has the highest nanofiber diameter and a high salt additive rate. The results obtained showed that low amounts of TCL salt were not toxic. It has even been reported that TCL salt has a supportive effect on fibroblast cell viability. However, a decrease in cell viability was observed with the high amount of TCL salt in the structure.

According to the ISO 10993‐5 standard, the usability of a material as a wound dressing is associated with a cell viability of 70% or above [1]. According to the cell viability results obtained in this study, it was suggested that all nonwoven networks can be used as wound dressings. It has also been reported that different addition ratios of TCL salt increased the cell viability of nanofibers in the range of 42.70% to 14.80% compared to the cell viability of pure PLA–PEG nanofibers. Accordingly, it is predicted that the use of the lowest TCL‐added PLA–3TCL–PEG nonwoven network as a dermal wound dressing will accelerate wound‐healing by contributing to the highest fibroblast cell proliferation.

**FIGURE 11:**
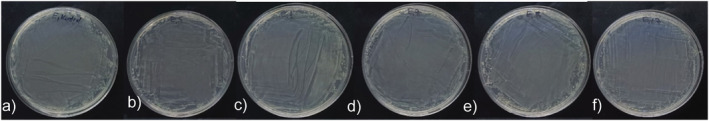
Plaque images showing the decrease caused by nonwoven webs in *E. coli* bacteria at the end of 24 h. (a) Control, (b) PLA–3TCL–PEG, (c) PLA–5TCL–PEG, (d) PLA–7TCL–PEG, (e) PLA–9TCL–PEG, and (f) PLA–PEG.

**FIGURE 12:**
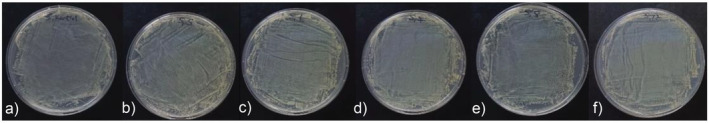
Plaque images showing the decrease caused by nonwoven webs in *S. aureus* bacteria at the end of 24 h. (a) Control, (b) PLA–3TCL–PEG, (c) PLA–5TCL–PEG, (d) PLA–7TCL–PEG, (e) PLA–9TCL–PEG, and (f) PLA–PEG.

The antibacterial activity (%) of pure PLA–PEG and PLA–TCL–PEG nonwoven networks against *E. coli* and *S. aureus* bacteria at 24 h and the number of bacteria on the plate are given in Table 4. *E. coli* and *S. aureus* bacteria observed on the plate after 24 h are given in Figures 11 and 12. According to the test results performed to evaluate the antibacterial activity of TCL salt, no antibacterial activity was observed against *E. coli* or *S. aureus* cultures. The reliability and consistency of the test were ensured with a bacteria control plate. Pure PLA–PEG nanofiber was also used as a control sample to evaluate the antibacterial activity among salt‐doped nanofibers. QAS are known to have antibacterial activity [70]. We have proven the antibacterial activity of benzalkonium chloride salt in our previous studies [71]. It was stated that the obtaining nonwoven webs did not have antibacterial activity. As a result, it was reported that TCL salt and different additive ratios had no effect on antibacterial activity behavior. However, the PLA–TCL–PEG nonwoven networks were obtained in this study.

**TABLE 4:** The antibacterial activity (%) of nonwoven webs against *E. coli* and *S. aureus*.

Samples	*E. coli* (0 h)	*E. coli* (24 h)	Antibacterial activity (%)	*S. aureus* (0 h)	*S. aureus* (24 h)	Antibacterial activity (%)
PLA–PEG	1,455,000	Average: ~10^8^ (uncountable)	—	475.000	Average: ~10^8^ (uncountable)	
PLA–3TCL–PEG	1,455,000	Average: ~10^8^ (uncountable)	—	475.000	Average: ~10^8^ (uncountable)	—
PLA–5TCL–PEG	1,455,000	Average: ~10^8^ (uncountable)	—	475.000	Average: ~10^8^ (uncountable)	—
PLA–7TCL–PEG	1,455,000	Average: ~10^8^ (uncountable)	—	475.000	Average: ~10^8^ (uncountable)	—
PLA–9TCL–PEG	1,455,000	Average: ~10^8^ (uncountable)	—	475.000	Average: ~10^8^ (uncountable)	—

## 4 Conclusions

In this study, electrospun mats were produced using PLA–PEG and tetrapropylammonium chloride quaternary ammonium salt. When the FTIR spectrum was examined, it was stated that there was no specific effect on the structure of PLA–TCL–PEG nonwoven webs with the addition of TCL salt, but it caused a decrease in some peak intensities. According to SEM micrografts, it was stated that with the addition of TCL salt, the beads seen in the PLA–PEG nonwoven webs disappeared; thus, the nanofiber surfaces were improved. While the most homogeneous and uniform nanofibers were obtained in the PLA–5TCL–PEG nonwoven web, it was reported that the PLA–3TCL–PEG web had the lowest average fiber diameter among all nonwoven webs. A significant increase in LAC percentages was observed with the addition of TCL salt. The PLA–5TCL–PEG web exhibited both the maximum LAC percentage and shortest drying time. Additionally, according to TGA results, thermal properties decreased with the addition of TCL salt, but mechanical properties increased with the % elongation value. According to the cytotoxicity test results, cell viability clearly increased with the addition of TCL salt to the structure. While maximum cell viability was observed in the PLA–3TCL–PEG nonwoven web, the values decreased with the increase of salt additives. Nevertheless, PLA–9TCL–PEG nanofibers resulted in a higher value than PLA–PEG nanofibers. When evaluated together with the SEM images and average fiber diameter results, it is related to these results. It has been reported that TCL salt does not provide antibacterial activity to PLA–PEG nonwoven webs.

## Author Contributions

**Sena Özdil Şener:** conducting experiments, review and editing, research, writing original drafts, visualizing data collection and analysis, sources, writing supervision, and literature review. **Sema Samatya Yilmaz:** experimental design, conducting experiments, data interpretation, research, project management, supervision of experiments, data improvement, writing – review, sources data collection, and analysis. **Merve Dandan Doganci** and **Erdinc Doganci:** project management, writing – review and editing, data curation, concept and design of work, conceptualization, visualization, data interpretation, sources, auditing, and literature review.

## Conflicts of Interest

The authors declare no conflicts of interest.

## Data Availability

The data that support the findings of this study are available from the corresponding author upon reasonable request.
